# Branched chain α‐ketoacid dehydrogenase kinase 111–130, a T cell epitope that induces both autoimmune myocarditis and hepatitis in A/J mice

**DOI:** 10.1002/iid3.177

**Published:** 2017-06-09

**Authors:** Bharathi Krishnan, Chandirasegaran Massilamany, Rakesh H. Basavalingappa, Arunakumar Gangaplara, Guobin Kang, Qingsheng Li, Francisco A. Uzal, Jennifer L. Strande, Gustavo A. Delhon, Jean‐Jack Riethoven, David Steffen, Jay Reddy

**Affiliations:** ^1^ School of Veterinary Medicine and Biomedical Sciences University of Nebraska‐Lincoln Lincoln Nebraska USA; ^2^ Laboratory of Immunology, National Institute of Allergy and Infectious Diseases National Institutes of Health Bethesda Maryland USA; ^3^ Nebraska Center for Virology, School of Biological Sciences University of Nebraska‐Lincoln Lincoln Nebraska USA; ^4^ School of Veterinary Medicine University of California Davis California USA; ^5^ Department of Medicine, Cell Biology, Neurobiology and Anatomy Medical College of Wisconsin Milwaukee Wisconsin USA; ^6^ Center for Biotechnology University of Nebraska‐Lincoln Lincoln Nebraska USA

**Keywords:** Autoimmune hepatitis, autoimmune myocarditis, autoreactive T cells, branched chain α‐ketoacid dehydrogenase kinase, mouse model

## Abstract

**Introduction:**

Organ‐specific autoimmune diseases are believed to result from immune responses generated against self‐antigens specific to each organ. However, when such responses target antigens expressed promiscuously in multiple tissues, then the immune‐mediated damage may be wide spread.

**Methods:**

In this report, we describe a mitochondrial protein, branched chain α‐ketoacid dehydrogenase kinase (BCKD_k_) that can act as a target autoantigen in the development of autoimmune inflammatory reactions in both heart and liver.

**Results:**

We demonstrate that BCKD_k_ protein contains at least nine immunodominant epitopes, three of which, BCKD_k_ 71–90, BCKD_k_ 111–130 and BCKD_k_ 141–160, were found to induce varying degrees of myocarditis in immunized mice. One of these, BCKD_k_ 111–130, could also induce hepatitis without affecting lungs, kidneys, skeletal muscles, and brain. In immunogenicity testing, all three peptides induced antigen‐specific T cell responses, as verified by proliferation assay and/or major histocompatibility complex class II/IA^k^ dextramer staining. Finally, the disease‐inducing abilities of BCKD_k_ peptides were correlated with the production of interferon‐γ, and the activated T cells could transfer disease to naive recipients.

**Conclusions:**

The disease induced by BCKD_k_ peptides could serve as a useful model to study the autoimmune events of inflammatory heart and liver diseases.

## Introduction

Various infectious and non‐infectious origins have been implicated in the causation of myocarditis. Many affected individuals recover spontaneously from myocarditis but, a proportion of patients can develop chronic inflammation leading to dilated cardiomyopathy (DCM) 1, 2. Mechanistically, autoimmune responses against cardiac antigens are believed to mediate the development of DCM, as sera from DCM patients have been shown to contain autoantibodies for cardiac antigens, such as cardiac myosin, and cardiac troponin‐I (cTnI) 3, 4, 5, 6, 7. Recently, we demonstrated that the mitochondrial inner membrane protein, adenine nucleotide translocator_1_ (ANT_1_) can be a target autoantigen in the pathogenesis of DCM by identifying ANT_1_ 21–40 as the disease‐inducing epitope in A/J mice 2. In this report, we describe the potential relevance of the mitochondrial matrix branched chain α‐ketoacid dehydrogenase (BCKD) complex proteins to myocarditis and hepatitis.

The BCKD complex includes three catalytic subunits (E1α and E1β, E2, and E3) and two regulatory proteins, BCKD kinase (BCKD_k_) and BCKD phosphatase, which bind noncovalently to the core protein E2 subunit 8, 9, 10, 11. These proteins can become targets for immune attack as demonstrated by the detection of BCKD‐reactive immune complexes in DCM patients and such a reactivity appears to be seen mostly in the E2 subunit 3, 12, 13. Whether other subunits of BCKD complex proteins can act as immune targets in the mediation of autoimmune myocarditis is unexplored. In our studies, we focused on BCKD_k_, the critical negative regulator of E1α subunit to determine its ability to induce myocarditis as its expression was found to be more in heart than other organs 14, 15, 16, 17. Additionally, existence of a single isomeric form of BCKD_k_ enabled us to generate overlapping peptide library to identify the immunodominant epitopes unique to this protein. We demonstrate that BCKD_k_ contains at least nine epitopes, three of which induce myocarditis in A/J mice by generating interferon‐γ (IFN‐γ) producing autoreactive T cells. One of these peptides also induces inflammation in the liver but other organs, lungs, kidneys, skeletal muscles, and brain remained unaffected.

## Results

### BCKD_k_ contains multiple epitopes that can induce myocarditis

The BCKD_k_ overlapping peptide library that we had generated consisted of 37 peptides (Supporting information Table S1). We used acetylated peptides as they are expected to prevent from intracellular degradation leading to better binding with major histocompatibility complex (MHC) molecules and better disease induction 2, 18, 19. For initial screening, eight pools were prepared to contain four to five peptides each, and the peptide/complete Freund's adjuvant (CFA) emulsions were administered to groups of mice. After three weeks, T cell responses were examined using bovine ribonuclease (RNase) 43–56 as a control. We noted that one or more peptides in each group induced significant dose‐dependent T cell responses (∼1.2 to threefold), leading us to identify a total of 23 peptides as potential immunodominant epitopes of BCKD_k_ (Supporting information Table S2). The responses were specific to BCKD_k_ peptides as the antigen‐sensitized cells did not respond to irrelevant control (RNase 43–56) peptide. Since our intent was to identify the disease‐inducing epitopes, we examined hearts for inflammatory changes by hematoxylin and eosin (H & E) staining. The data revealed that hearts obtained from groups II, III, and VIII showed mild myocarditis, but heart sections derived from other groups remained unaffected (data not shown).

**Figure 1:**
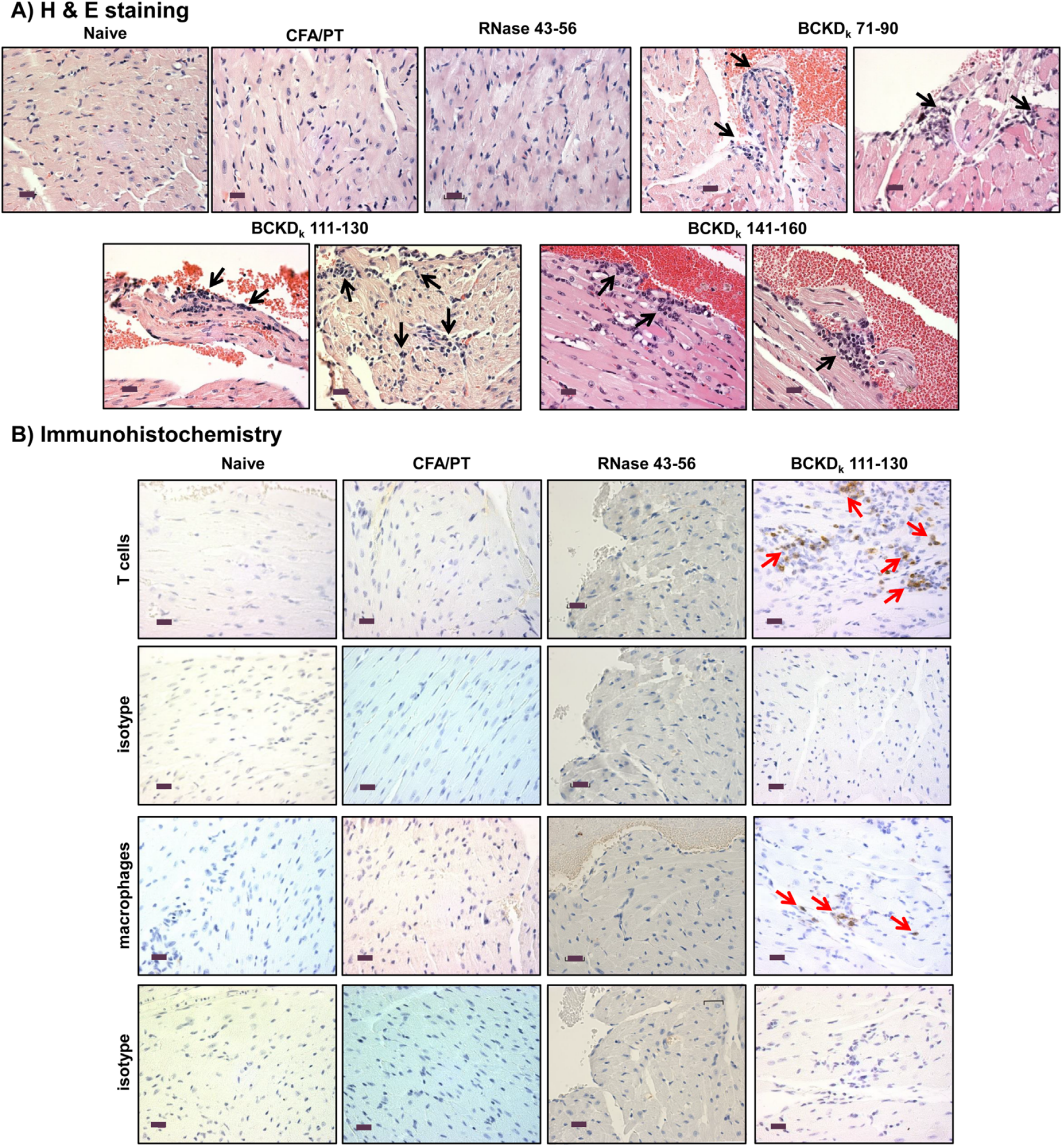
Induction of myocarditis by BCKD_k_ peptides in A/J mice. At termination on day 21 postimmunizations, hearts were collected for histological evaluation. (A) H & E staining. Inflammatory foci are shown with arrows in sections representing each group. Controls (naive, CFA/PT and RNase 43–56): normal cardiac tissue with intact myocytes; BCKD_k_ 71–90: moderate inflammatory foci in the endocardium; BCKD_k_ 111–130: multiple inflammatory foci in the endocardium (left panel) and myocardium (right panel); and BCKD_k_ 141–160: multiple foci in the endocardium. (Controls: *n* = 5 mice; BCKD_k_ 71–90, BCKD_k_ 111–130, and BCKD_k_ 141–160: *n* = 5 mice/group from two independent experiments). (B) IHC. Heart sections obtained from controls (naive, CFA/PT and RNase 43–56) and BCKD_k_ 111–130 groups were stained with antibodies for T cells (anti‐CD3) and macrophages (anti‐CD11b) or their isotype controls to detect cells positive for each marker shown (arrows). No CD3^+^ and CD11b^+^ cells were detected in control groups but were detected in the sections derived from BCKD_k_ 111‐130‐immunized animals (arrows). (Controls: *n* = 5 mice each; BCKD_k_ 111–130: *n* = 4 mice). Original magnification ×400 (scale bar: 20 µm).

To test their ability to induce myocarditis individually, we selected a total of nine peptides, three from each of groups II (BCKD_k_ 61–80, BCKD_k_ 71–90 and BCKD_k_ 91–110), III (BCKD_k_ 111–130, BCKD_k_ 121–140, and BCKD_k_ 141–160) and VIII (BCKD_k_ 331–350, BCKD_k_ 341–360, and BCKD_k_ 351–370) (Supporting information Table S2). Three peptides—BCKD_k_ 111–130, BCKD_k_ 71–90 and BCKD_k_ 141–160—were found to induce myocarditis in a descending order (7/10, 5/10, and 2/10), and the number of inflammatory foci in the respective groups were 5.29 ± 2.67, 2.20 ± 0.97, and 7.50 ± 6.50 (Table 1). The control groups (naive, CFA/pertussis toxin [PT] and RNase 43–56) lacked inflammatory changes (Table 1). Both endocardium and myocardium were found to be affected with a tendency for lesions to be seen more in the BCKD_k_ 111–130 group than the other two peptides (BCKD_k_ 71–90 and BCKD_k_ 141–160) (Fig. 1A). Immunohistochemistry (IHC) on heart sections of BCKD_k_ 111‐130‐immunized group, also stained positive for CD3^+^ T cells and CD11b^+^ macrophages, the two common leukocyte subsets expected in T cell‐mediated inflammatory reaction (Fig. 1B). The staining with isotype controls was negative, and none of the sections were stained positive for granulocytes as determined by staining with Ly6G antibody (data not shown). Similarly, the control groups (naive, CFA/PT and RNase 43–56) also lacked CD3^+^ and CD11b^+^ cells (Fig. 1B). Thus, we identified BCKD_k_ 111–130 as the primary myocarditogenic epitope in A/J mice.

**Table 1:** Histological evaluation of hearts obtained from mice immunized with BCKD_k_ peptides

Groups	Incidence (%)	Inflammatory foci (mean ± SEM)
Naive	0/5 (0)	0
CFA/PT	0/5 (0)	0
RNase 43–56	0/5 (0)	0
BCKD_k_ 61–80	0/5 (0)	0
BCKD_k_ 71–90	5/10 (50)	2.20 ± 0.97*
BCKD_k_ 91k110	0/5 (0)	0
BCKD_k_ 111k130	7/10 (70)	5.29 ± 2.67*
BCKD_k_ 121–140	0/5 (0)	0
BCKD_k_ 141–160	2/10 (20)	7.50 ± 6.50
BCKD_k_ 331–350	0/5 (0)	0
BCKD_k_ 341–360	0/5 (0)	0

*p < 0.05 vs. naive, CFA/PT and RNase 43–56 group.

### Echocardiographic analysis reveals cardiac abnormalities in animals immunized with‐ BCKD_k_ 111‐130

Echocardiograms of animals immunized with BCKD_k_ 111–130 revealed that the interventricular septal thickness at end‐diastole was increased compared to naive mice (0.93 ± 0.03 mm vs. 0.70 ± 0.06 mm; *p *< 0.05), whereas the left ventricular (LV) internal diameter at diastole remained unaltered (Supporting information Table S3). Consistent with this finding, heart weight and heart weight‐to‐body weight ratios also were increased in animals immunized with BCKD_k_ 111–130 (Supporting information Fig. S1). Functionally, although end diastolic and end systolic volumes were reduced in the BCKD_k_ 111‐130‐immunized group, differences were not significant. The data suggest BCKD_k_ 111–130 could induce structural changes in the hearts of myocarditic mice.

### BCKD_k_ 111–130 can induce autoimmune hepatitis in immunized mice

**Figure 2:**
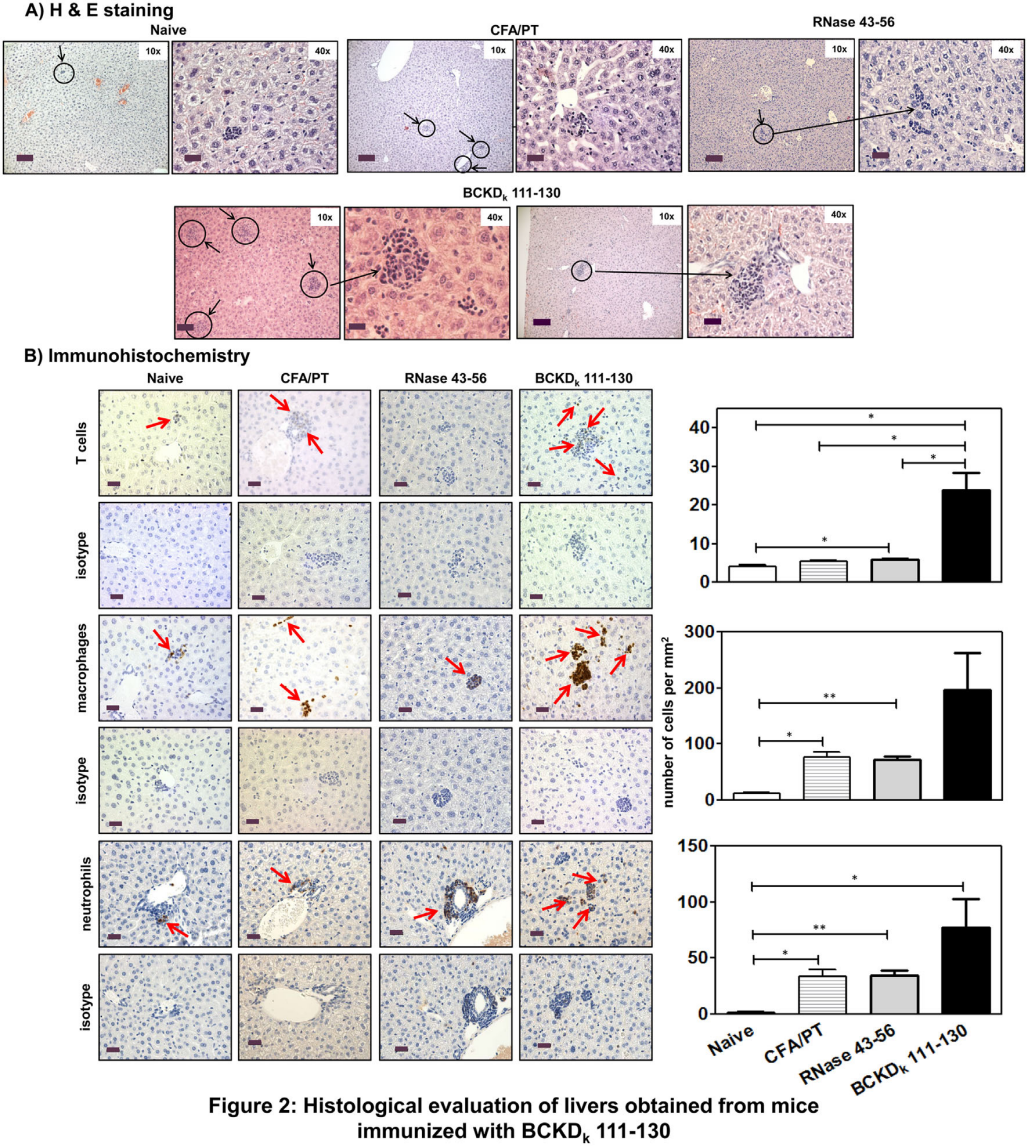
Histological evaluation of livers obtained from mice immunized with BCKD_k_ 111–130. Livers harvested from naive or day 21 postimmunized animals were examined for inflammatory changes. (A) H & E staining. Liver sections obtained from naive animals had a small aggregate of lymphocytes, as opposed to multiple scattered aggregates of mononuclear cells in animals immunized with CFA/PT and RNase 43–56, and similar changes, but with more pronounced lesions/large aggregates of MNCs in the parenchyma, and also in the periportal (centroacinar) region, in animals immunized with BCKD_k_ 111–130 (circles, 10× magnifications, scale bars: 80 µm; horizontal arrows, 40× magnifications, scale bars: 20 µm). (Controls: *n* = 5 mice each; BCKD_k_ 111–130: *n* = 5 mice/group from two independent experiments). (B) IHC. Liver sections revealed isolated/scattered CD3^+^ cells in naive, CFA/PT and RNase 43–56 groups that were elevated in animals immunized with BCKD_k_ 111–130 (top panel: arrows and bar graph). Similar patterns with CD11b^+^ and Ly6G^+^ cells were noted between treatment groups (middle and bottom panels: arrows and bar graphs). Mean ± SEM values are shown in bar graphs (CD3: *n* = 3 mice/group; CD11b: *n* = 4 mice/group and Ly6G^+^: *n* = 4 mice/group). Original magnification ×400 (scale bar: 20 µm). The *p* values were determined using Student's *t*‐test (*p ≤ 0.05 and **p ≤ 0.001).

Since BCKD_k_ is also expressed in non‐cardiac tissues such as liver, lungs, kidneys, skeletal muscles, and brain, we determined whether BCKD_k_ peptides could induce inflammation in multiple organs. Livers obtained from naive mice showed the presence of scattered inflammatory cell foci (1.80 ± 0.58), and, as expected, their numbers were significantly increased in those derived from the CFA/PT group (21.89 ± 2.75;*p *< 0.005) as we and others have previously described 2, 20. By using the CFA/PT group as a baseline, we compared the inflammatory cell foci detected in animals immunized with BCKD_k_ peptides, leading us to note that the livers from only the BCKD_k_ 111‐130‐immunized group showed a significant increase in the number of foci with leukocytes scattered all through the parenchyma (69.59 ± 8.98; *p *< 0.005), as compared to naive or CFA/PT groups (Table 2). Such an increase was not noted in animals immunized with a control peptide, RNase 43–56 (11.20 ± 1.83) suggesting that the inflammatory changes noted in BCKD_k_ 111‐130‐immunized animals were specific to antigen (Table 2). Histologically, foci were larger in animals immunized with BCKD_k_ 111–130 than in the control groups (naive, CFA/PT, and RNase 43–56) and the lesions comprising of lymphocytes and a few plasma cells and neutrophils were noted in the periportal (centroacinar) and midzonal areas (Fig. 2A). While similar trends were observed in three groups— BCKD_k_ 121–140, BCKD_k_ 141–160, and BCKD_k_ 331‐350—(Table 2) the numbers of liver foci were reduced in two other groups (BCKD_k_ 61–80: 10.54 ± 1.64 and BCKD_k_ 341–360: 13.24 ± 1.50). However, no such variations were noted in the remaining groups (BCKD_k_ 71–90, BCKD_k_ 91–110, and BCKD_k_ 351–370). Further, evaluation of other tissues such as lung, kidney, skeletal muscle and brain, derived from animals immunized with BCKD_k_ 71–90, BCKD_k_ 111–130 and BCKD_k_ 141–160 did not reveal any leukocyte infiltrates (data not shown). Since inflammatory foci in livers were increased selectively in animals immunized with BCKD_k_ 111–130, we enumerated the numbers of CD3^+^ T cells, CD11b^+^ macrophages and Ly6G^+^ neutrophils by IHC. These analyses revealed T cell numbers to be significantly higher in liver sections from BCKD_k_ 111‐130‐immunized animals (23.79 ± 4.57) than both CFA/PT (5.35 ± 0.22), RNase 43–56 (5.81 ± 0.24) and naive groups (4.15 ± 0.37) (Fig. 2B, top right panel). Although, similar trends were noted with CD11b^+^ and Ly6G^+^ cells, the differences were not significant between BCKD_k_ 111‐130‐immunized animals and control groups (CFA/PT and RNase 43–56) (CD11b^+^: Fig. 2B, middle right panel and Ly6G^+^ cells: Fig. 2B, bottom right panel). Taken together, the data suggest that BCKD_k_ 111–130 has a potential to induce autoimmune hepatitis in the immunized animals.

**Table 2:** Histological evaluation of livers obtained from mice immunized with BCKD_k_ peptides

Groups	Incidence (%)	Inflammatory foci (mean ± SEM)
Naive	5/5 (100)	1.80 ± 0.58
CFA/PT	5/5 (100)	21.89 ± 2.75**
RNase 43–56	5/5 (100)	11.20 ± 1.83
BCKD_k_ 61–80	5/5 (100)	10.54 ± 1.64
BCKD_k_ 71–90	10/10 (100)	19.99 ± 3.44
BCKD_k_ 91–110	5/5 (100)	20.23 ± 5.96
BCKD_k_ 111–130	10/10 (100)	69.59 ± 8.98**
BCKD_k_ 121–140	5/5 (100)	32.05 ± 10.88
BCKD_k_ 141–160	10/10 (100)	37.62 ± 7.17
BCKD_k_ 331–350	5/5 (100)	49.45 ± 21.74
BCKD_k_ 341–360	5/5 (100)	13.24 ± 1.50
BCKD_k_ 351–370	5/5 (100)	25.94 ± 5.43

**p < 0.005 vs. naive and/or CFA/PT group.

### Both pathogenic and nonpathogenic epitopes of BCKD_k_ can induce T cell responses in vitro

**Figure 3:**
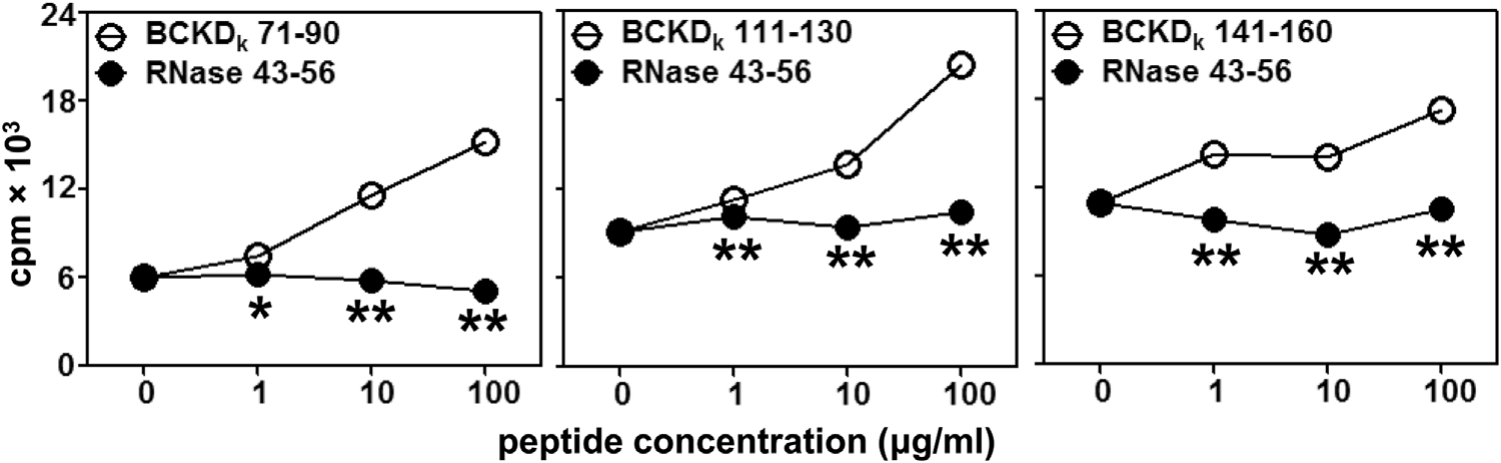
T cell responses induced by myocarditogenic BCKD_k_ peptides. LNCs prepared from immunized animals were stimulated with the immunizing peptides or RNase 43–56 (control) for 2 days, cells were pulsed with tritiated‐thymidine for 16 h, and proliferative responses were measured as counts per minute (cpm). Mean ± SEM values obtained from three individual experiments, each representing two to three mice, are shown. The *p* values were determined using Student's t‐test (*p < 0.05 and **p < 0.001 between indicated doses).

We performed T cell proliferation assay using a panel of nine peptides. We first verified that all the three myocarditogenic epitopes namely, BCKD_k_ 71–90, BCKD_k_ 111–130, and BCKD_k_ 141–160 induce specific T cell responses since the corresponding antigen‐sensitized cells did not respond to control (RNase 43–56) peptide (Fig. 3). The dose‐dependent differences (up to threefold) were comparable between all the three peptides, and the responses were significant relative to control suggesting that their disease‐inducing abilities may involve the mediation of autoreactive T cells. Next, in a similar analysis for six other peptides that failed to induce myocarditis or hepatitis, we noted that all of them induced significant proliferative responses when compared to RNase 43–56 (Supporting information Fig. S2). The finding that multiple BCKD_k_ epitopes could generate T cell responses regardless of their ability to induce disease implies that additional factors may be critical for epitopes to induce pathology. Finally, to determine whether unimmunized animals carry BCKD_k_‐reactive T cells in their naive repertoires, we analyzed T cell responses using lymphocytes obtained from naive A/J mice. Supporting information Figure S3 did not reveal detectable repertoires of T cells that react with any of the three peptides tested (BCKD_k_ 71–90, BCKD_k_ 111–130, and BCKD_k_ 141–160), suggesting that the occurrence of myocarditis and/or hepatitis induced by BCKD_k_ peptides does not involve the expansion of the preexisting peripheral repertoires of naive mice.

### BCKD_k_ epitopes can bind MHC class II allele, IA^k^ in A/J mice

**Figure 4:**
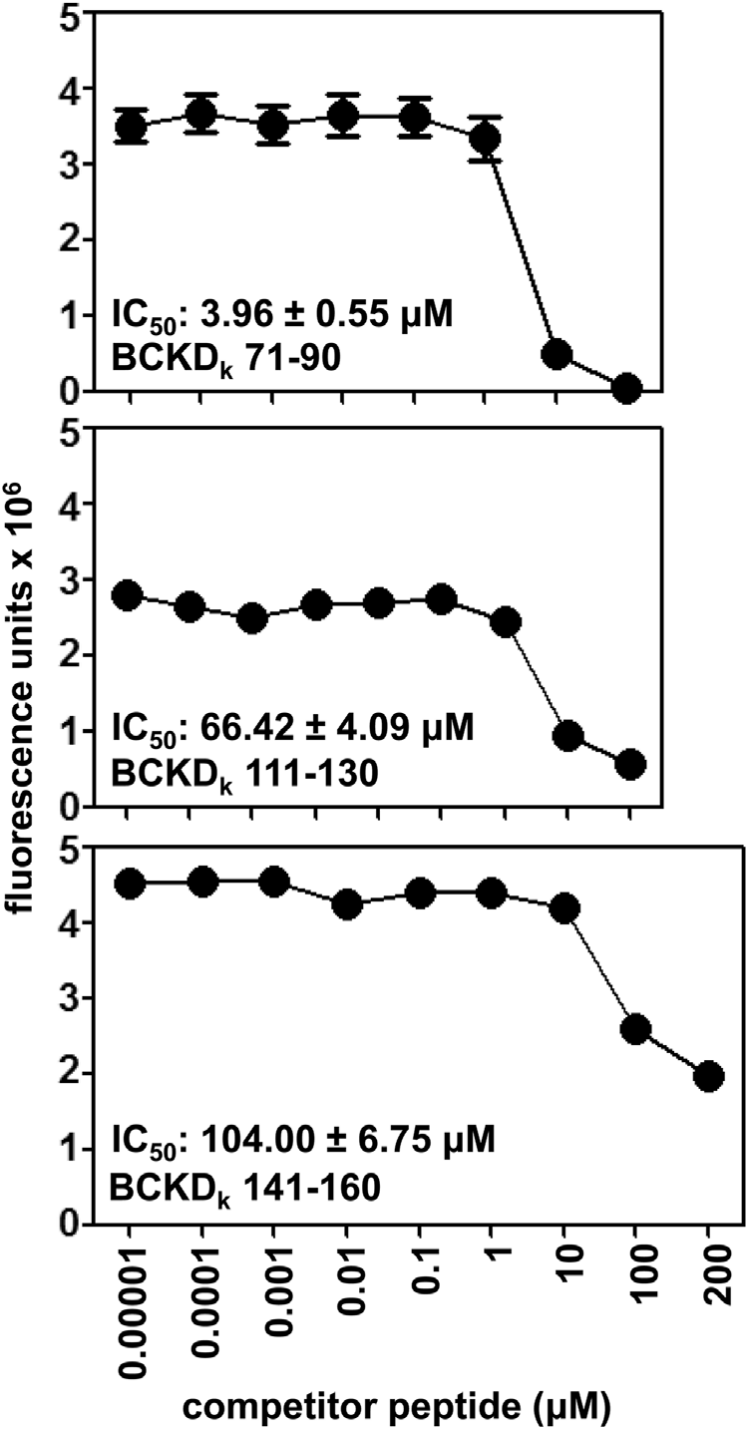
Evaluation of binding affinities of BCKD_k_ peptides to MHC class II/IA^k^ molecules. The reaction mixtures containing soluble, IA^k^ monomers, competitor peptides (BCKD_k_ 71–90, BCKD_k_ 111–130, BCKD_k_ 141–160: 0.00001–200 μM), and biotinylated hen egg lysozyme 46–61 (reference peptide; 1 μM) were prepared individually, after the incubation the reaction mixture were transferred to fluorescence plates precoated with IA^k^ antibody in duplicates. After a series of washes, europium‐labeled streptavidin was added to DELFIA buffer followed by DELFIA enhancer, and the fluorescence intensities were measured at excitation/emission wavelengths of 340/615 nm to obtain the IC_50_ values (*n* = 3).

Using the soluble empty IA^k^ molecules 21, we determined the 50% inhibitory concentration (IC_50_) values for the three test peptides that induced myocarditis and/or hepatitis. As shown in Figure 4, BCKD_k_ 71–90 was found to be a strong binder of IA^k^ molecules followed by BCKD_k_ 111–130, and BCKD_k_ 141–160. Their respective IC_50_ values were 3.96 ± 0.55 μM, 66.42 ± 4.09 μM, and 104.00 ± 6.75 μM. However, it is to be noted that A/J mice also express IE^k^ as another MHC class II allele, and there exists the possibility that BCKD_k_ 141–160 can bind the IE^k^ molecule, which we have not investigated in this study.

### The creation of IA^k^ dextramers permits evaluation of antigen‐specificity of BCKD_k_‐reactive T cells

**Figure 5:**
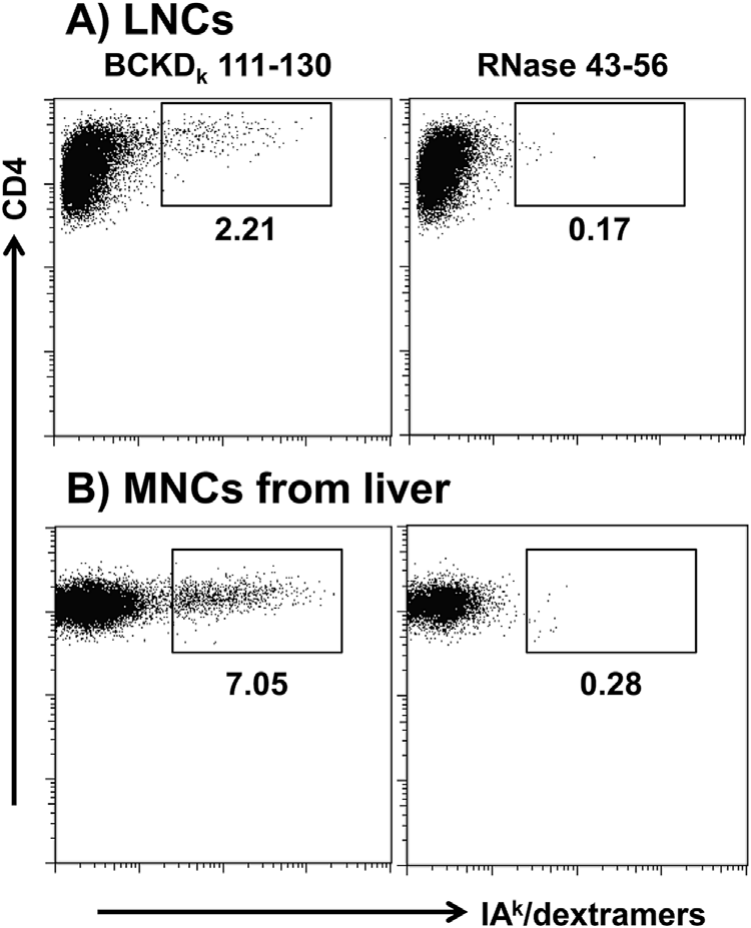
Antigen‐specificity of T cell responses induced by BCKD_k_ 111–130. (A) LNCs. Viable cells harvested from BCKD_k_ 111‐130‐reactive LNC cultures on day 9 poststimulation were stained with the indicated IA^k^ dextramers, anti‐CD4 and 7‐aminoactinomycin D. After acquiring the cells by flow cytometry, dextramer^+^ cells were analyzed in the live (7‐aminoactinomycin D^−^), CD4 subset. (B) MNCs from liver. Livers from immunized animals were processed to obtain MNCs as described in the methods section. After stimulating the cells with BCKD_k_ 111–130 and resting in IL‐2 medium, cells were subjected for dextramer staining on day 8 and the dextramer^+^ cells were determined as above. RNase 43–56 dextramers (control). Representative flow cytometric plots from three individual experiments each involving two to three mice are shown.

To validate dextramers, we used lymph node cell (LNC) cultures derived from BCKD_k_ 111‐130‐immunized animals as described previously 22. Cells harvested from antigen‐stimulated cultures were harvested on day 9 poststimulation with BCKD_k_ 111–130, and the cells were stained with BCKD_k_ 111–130 and control (RNase 43–56) dextramers. The flow cytometric plots depicted in Figure 5A indicate that the CD4 T cells were stained with BCKD_k_ 111–130 dextramers, whereas the intensity of staining obtained with the control dextramers was negligible (2.21% vs. 0.17%). Thus, we demonstrated that T cell response induced with BCKD_k_ 111–130 was antigen‐specific. We next asked whether antigen‐specific T cells can infiltrate into the target organs. Since, liver being a large organ, we were able to harvest mononuclear cells (MNCs) from immunized animals that permitted us to perform dextramer staining by flow cytometry. MNCs were stimulated with BCKD_k_ 111–130 and on day 8 postimulation, cells were subjected for dextramer staining. The analysis revealed detection of BCKD_k_ 111–130 CD4^+^dextramer^+^ T cells, but not control dextramers (7.05% vs. 0.28%) (Fig. 5B) suggesting infiltration of antigen‐specific T cells into the livers. Since animals were perfused prior to harvesting MNCs from livers, it is unlikely that BCKD_k_ 111–130‐reactive T cells might have represented the peripheral blood compartment. Nonetheless, while flow cytometric analysis permits enumeration of total number of antigen‐specific T cells, determination of their localization requires establishment of in situ staining technique and we have not investigated this possibility.

### Cytokine responses induced by BCKD_k_ 111–130 were predominantly of T helper (Th) 1 phenotype

**Figure 6:**
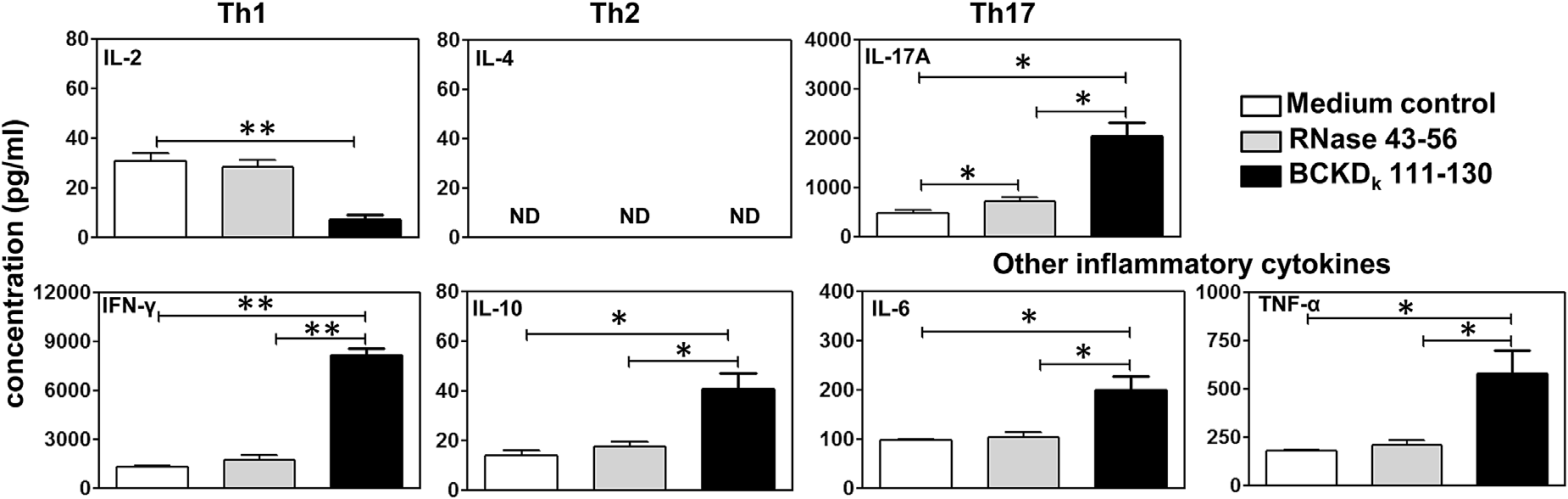
Cytokine responses induced by BCKD_k_ 111–130. LNCs obtained from immunized mice were stimulated with or without BCKD_k_ 111–130 or RNase 43–56 (control). Supernatants collected on day 3 poststimulation were examined by cytometric bead array analysis. Mean ± SEM values obtained from three individual experiments are shown. The *p* values were determined using Student's *t*‐test (*p < 0.05 and **p < 0.005). ND; not detectable.

Culture supernatants from LNC cultures stimulated with or without BCKD_k_ 111–130 or irrelevant control (RNase 43–56) were subjected for cytokine analysis. The data revealed that all groups showed the detection of all cytokines tested except interleukin (IL)‐4 (Fig. 6), but their patterns varied. First, BCKD_k_ 111‐130‐induced cytokine responses included all three phenotypes—Th1 (IFN‐γ), Th2 (IL‐10), and Th17 (IL‐17A)—including two other prototypical inflammatory cytokines (IL‐6 and tumor necrosis factor (TNF)‐α). Their levels were significantly higher than the control groups. Second, production of IL‐17A in RNase 43–56‐stimulated cultures also tended to be greater than the medium controls, implying that IL‐17A production could have been triggered non‐antigen‐specifically. Third, IL‐2 production was significantly lower in cultures stimulated with BCKD_k_ 111–130, but not in RNase 43‐56‐stimulated cultures, indicating that the antigen‐responsive T cells consume IL‐2 as it is secreted. Fourth, since production of IFN‐γ was highest compared to other cytokines, we determined the ratios between IFN‐γ (Th1) to IL‐17A (Th17), IL‐10 (Th2), IL‐6, and TNF‐α to be 4, 201, 41, and 14‐fold, respectively. Similar patterns also were observed for one other myocarditogenic epitope, BCKD_k_ 71–90, for which supernatants of cultures obtained from animals immunized for disease induction were evaluated for cytokine analysis (Supporting information Fig. S4A). Under similar conditions, however, inflammatory cytokines produced by a weak myocarditogenic epitope (BCKD_k_ 141‐160; supporting information Fig. S4B) did not differ significantly, indicating correlation between production of inflammatory cytokines and disease severity. We conclude that eventhough production of IL‐10, an anti‐inflammatory cytokine, is increased in BCKD_k_ 111‐130‐stimulated cultures, such production was significantly lower (40.79 ± 6.51 pg/ml) than the sum production of all other inflammatory cytokines (IFN‐γ, 8195.11 ± 416.56 pg/ml; IL‐17A, 2036.30 ± 290.86 pg/ml; IL‐6, 200.22 ± 28.15 pg/ml; and TNF‐α, 582.81 ± 116.72 pg/ml), negating the anti‐inflammatory effects of IL‐10, leading to the induction of inflammation in the target organs.

### BCKD_k_ 111‐130‐sensitized T cells can transfer disease to naive animals

**Figure 7:**
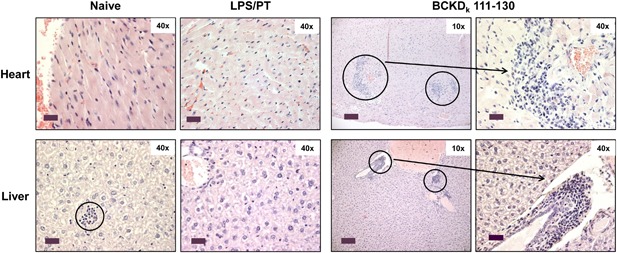
Disease induction by BCKD_k_ 111‐130‐sensitized T cells in naive mice. LNCs prepared from animals immunized with BCKD_k_ 111–130 were stimulated with concanavalin‐A for 2 days. Viable cells were administered into naive mice primed with LPS, and naive and LPS/PT‐alone groups were used as controls. At termination on day 14, hearts and livers were collected for H & E staining and representative sections are shown from two individual experiments (*n* = 2/group). Top panel: normal cardiac tissues in naive and LPS/PT groups and multiple inflammatory foci in the myocardium from the BCKD_k_ 111–130 group are depicted. Bottom panel: naive mice had isolated foci in their livers; LPS/PT group showed no detectable lesions, whereas prominent foci in the periportal (centroacinar) areas were detected in animals infused with BCKD_k_ 111–130 (circles, 10× magnifications, scale bars: 80 µm; horizontal arrows, 40× magnifications, scale bars: 20 µm). Original magnification ×400 (Scale bar: 20 µm).

To evaluate the disease‐inducing abilities of autoreactive T cells, we used an adoptive transfer protocol to induce disease in naive mice 2, 23, 24. Heart sections obtained from recipients of BCKD_k_ 111‐130‐reactive T cells showed multiple inflammatory foci in the myocardium (6.70 ± 2.40) and rarely necrosis, both of which were absent in the control groups (naive and lipopolysaccharide [LPS] primed; Fig. 7, top panel). In a similar analysis, other tissues, except liver, remained unaffected (data not shown); the number of inflammatory foci in livers from recipients of BCKD_k_ 111‐130‐senstized T cells was estimated to be higher and larger (7.77 ± 1.31) than those in naive (1.80 ± 1.00) or LPS/PT groups (0.25 ± 0.25), and the lesions were localized mostly to periportal (centroacinar) areas (Fig. 7, bottom panel). These data complement the observations made in the active immunization protocol as described above.

## Discussion

In this report, we demonstrate that BCKD_k_, a mitochondrial protein, contains multiple epitopes that induce varying degrees of inflammatory reactions in heart and/or liver in A/J mice. We were able to identify three peptides that are capable of inducing myocarditis—BCKD_k_ 71–90, BCKD_k_ 111–130 and BCKD_k_ 141–160—and BCKD_k_ 111–130 being the efficient. All three peptides induced similar inflammatory phenotypes without associated necrosis. BCKD_k_ 111‐130‐induced inflammatory lesion contained both T cells and macrophages, suggesting that the disease pathogenesis involves the mediation of classic delayed hypersensitivity reaction as expected in T cell‐mediated autoimmune diseases 25. Since most immunized animals showed mild multifocal lesions, the inflammatory damage induced by BCKD_k_ peptides might not be severe enough to cause necrotic changes as seen with other experimental autoimmune myocarditis (EAM) models like cardiac myosin 19, 21 and cTnI 7, 26, 27. Nonetheless, this degree of myocardial inflammation caused regional increases in LV wall thickness as detected by echocardiography, but other functional changes such as end‐diastolic volume and end‐systolic volume were not significantly altered, most likely reflecting the lack of necrosis in these hearts. Taken together, our data suggest that BCKD_k_, in spite of being a mitochondrial protein, can become a target for immune attack resulting in autoimmune reactions in the heart. We also noted an autoimmune response in the liver, as inflammatory foci were significantly elevated in livers from only BCKD_k_ 111–130‐immunized group as compared to control groups (naive, CFA/PT, and RNase 43–56). The lesions were also noted in the periportal (centroacinar) areas as noted in the mouse model of autoimmune hepatitis 28. It should be noted, however, that animals receiving CFA/PT can show elevations in the inflammatory foci in the liver resulting from non‐specific effects of the adjuvant, as we and others have reported previously 2, 20. Nonetheless, the finding that the number of liver foci were increased only in animals immunized with BCKD_k_ 111–130 but not the other eight peptides and also an irrelevant antigen (RNase 43–56) (Table 2) suggests that BCKD_k_ 111–130 also can be an autoantigen in the mediation of autoimmune hepatitis. However, none of the three peptides tested (BCKD_k_ 71–90, BCKD_k_ 111–130, and BCKD_k_ 141–160) were found to induce inflammation in lungs, kidneys, skeletal muscles, and brain (data not shown). Thus, we have identified BCKD_k_ 111–130 as a major epitope that can induce autoimmune inflammatory reactions in both heart and liver in A/J mice.

We next investigated whether the disease induced with BCKD_k_ peptides can be correlated with T cell responses. As expected, the three peptides, BCKD_k_ 71–90, BCKD_k_ 111–130, and BCKD_k_ 141–160 that induced myocarditis were also strong activators of T cells. In corollary, those peptides (BCKD_k_ 331–350 or BCKD_k_ 341–360) that were poor T cell activators were not able to induce disease. However, not all the peptides that activated T cells could induce disease for example, BCKD_k_ 121–140 (Supporting information Fig. S2; Table 1) supporting the notion that appearance of autoreactive T cells does not necessarily lead to pathogenic outcomes. Further, by determining the cytokine responses, we noted that BCKD_k_ 111–130‐senstitized cultures showed the predominant levels of IFN‐γ, followed by IL‐17A and other inflammatory cytokines (TNF‐α and IL‐6) (Fig. 6). While these results agree with the expected cytokine pattern as seen in other EAM models such as cardiac myosin, cTnI, and ANT_1_ 2, 26, 27, 29, 30, we also found elevated levels of the anti‐inflammatory cytokine IL‐10. However, IL‐10 at a ∼200‐fold decreased level than that of IFN‐γ alone suggests that eventhough both pro‐ and anti‐inflammatory cytokine responses occur with BCKD_k_, the balance is shifted in favor of the pro‐inflammatory state. Additionally, in an adoptive transfer protocol, we demonstrated that BCKD_k_ 111–130‐sensitized T cells can transfer disease to naive mice as the recipients showed both myocardial and liver lesions. We have consistently seen that hearts from control groups (naive and LPS/PT) do not show inflammatory foci 21, 24. As to livers, however, scattered inflammatory foci were present as expected in both control groups, but their detection was significantly enhanced in the recipients of BCKD_k_ 111–130‐reactive T cells suggesting a role for antigen‐specific T cells in the development of hepatitis. Detection of BCKD_k_ 111‐130‐dextramer^+^ T cells in livers from immunized animals also supports this possibility. However, it is to be noted that local inflammatory environment in the liver can promote recruitment of autoreactive T cells that can potentially induce bystander hepatitis, and T cells can undergo apoptosis within the liver 31. Thus, the presence of autoreactive T cells may not necessarily lead to liver injury.

In summary, we have shown that mitochondrial proteins like BCKD_k_ can become immune targets in the mediation of autoimmune myocarditis and/or hepatitis by generating autoreactive T cells. It is unexpected that mitochondria which are sub‐cellular organelles can give rise to proteins capable of generating an autoimmune response. However, other mitochondrial proteins have been documented to play a role in autoimmune pathology including the E2 subunit of pyruvate dehydrogenase, which has been shown to act as a target antigen in the mediation of primary biliary cirrhosis 32, 33 and ANT_1_ 21–40 that we have previously shown to induce myocarditis in a manner similar to BCKD_k_ peptides 2. Our studies raise questions that how the mitochondrial proteins could be presented specifically to autoreactive CD4 T cells to induce disease in heart and liver. We speculate that the resident antigen‐presenting cells (APCs) uptake and display, the immunodominant epitopes to autoreactive T cells. Still, it is unknown whether resident APCs in target organs like heart and liver display BCKD_k_ fragments as reported for cardiac myosin in previous studies, in which APCs expressing myosin fragment were demonstrated in hearts from A/J mice 6, 12. The heart is a mitochondrial‐rich organ, and misfolded mitochondrial proteins if any, may be shuttled to the proteasomal degradation. Alternatively, damaged mitochondria can undergo degradation and they can be removed from cardiomyocytes by a process called, mitophagy 34. It is possible that either of these two mechanisms can result in immunogenic mitochondrial protein epitopes being released from the cardiomyocytes to be taken up by resident APCs. Additionally, in response to stressful microenvironment, cells undergoing apoptosis may also be engulfed by APCs as a part of a clean‐up process. Our data provide experimental support for an observation made in humans that the antibody reactivity to BCKD complex proteins in DCM patients can possibly involve the generation of BCKD_k_‐reactive T cells, since T cell help through cytokines is critical for antibody production. Additionally, we noted that humans can express four isoforms of BCKD_k_ as opposed to mice, where no isoforms were reported. The gene structure and amino acid sequence of BCKD_k_ are highly conserved between mice and humans with 94.5% identity. Comparison of sequences of BCKD_k_ peptides between humans and mice revealed only a single amino acid to be different with respect to BCKD_k_ 71–90 and BCKD_k_ 141–160, whereas the sequence of BCKD_k_ 111–130 was fully identical in both the species. Whether BCKD_k_ 111–130 can also act as an immunodominant T cell epitope in humans is currently unknown. Proving this to be true may permit evaluation of BCKD_k_‐reactive T cell responses in patients with DCM in clinical settings since BCKD‐reactive immune complexes have been demonstrated in DCM patients 3, 12, 13. It may be possible that cardiac damage resulting from infectious or noninfectious causes can lead to the release of cytoplasmic proteins like BCKD_k_ to the extracellular milieu. Such antigens can potentially trigger autoimmune responses and contribute to cardiac pathology.

## Materials and Methods

### Ethics statement

A/J mice (6‐ to 8‐week‐old, female, H‐2^a^) obtained from the Jackson Laboratory (Bar Harbor, ME) and approval for animal studies was granted by the Institutional Animal Care and Use Committee (IACUC), University of Nebraska‐Lincoln, Lincoln, NE; IACUC protocol #: 1398. At termination, animals were euthanized using a carbon dioxide chamber according to the Panel on Euthanasia, the American Veterinary Medical Association.

### Peptide synthesis

A total of 37 acetylated peptides containing acetyl group at the N‐terminal end (20‐mers with an overlap of 10 amino acids) representing 380 residues of mouse BCKD_k_ excluding the mitochondrial signaling sequence of 32 amino acids, RNase 43–56 and biotinylated hen egg lysozyme 46–61 20 were used in this study were synthesized on 9‐fluorenylmethyloxycarbonyl chemistry. All peptides were high‐performance liquid chromatography‐purified (>90%), and their identity was confirmed by mass spectroscopy (Neopeptide, Cambridge, MA). The peptides were dissolved in ultra‐pure water, aliquoted, and stored at −20°C.

### Immunization procedures

Peptide emulsions were prepared in CFA containing *Mycobacterium tuberculosis* H37RA extract (5 mg/ml, Difco Laboratories, Detroit, MI). Mice were immunized with these emulsions subcutaneously on days 0 and 7. Animals also received PT (List Biological Laboratories, Campbell, CA; 100 ng/mouse) intraperitoneally (i.p.) on days 0 and 2 after the first immunization 21. In pooled settings, four to five peptides of 50 μg each were mixed together, whereas mice immunized with individual peptides received 100 µg in each injection. For MHC class II dextramer staining and cytokine analysis for BCKD_k_ 111–130, animals received only one dose of peptide emulsions. Mice that received CFA/PT alone served as controls. These animals were administered with CFA emulsion (day 0 and 7), and PT (day 0 and 2). As an additional control group, animals were immunized with RNase 43–56 as an irrelevant control antigen in CFA on day 0 and 7, and PT was administered on day 0 and 2 after the first immunization i.p.

### T cell proliferation assay

LNCs obtained from animals on day 21 postimmunization were used to assess their proliferative responses based on tritiated‐thymidine‐incorporation assay. The proliferative responses were measured as counts per minute (cpm) 21, 22. For easy depiction, where indicated, T cell responses are shown as fold changes derived by dividing the cpm values of cultures stimulated with peptides by the cpm values of unstimulated cultures (medium controls) 2.

### H & E staining

Tissues (heart, liver, lung, kidney, skeletal muscle and brain) were collected at termination on day 21, fixed in 10% phosphate‐buffered formalin and processed for the production of 5 μm thick H & E serial sections, obtained ∼50 μm apart from each other. All sections were examined by a board‐certified pathologist blinded to treatment. The total number of inflammatory cell foci was determined as reported previously 21, 30, 35. For evaluation of inflammatory foci in the livers, stained sections were scanned with the aid of Aperio digital pathology slide scanners (Leica Biosystems, Wetzlar, Germany). After counting the foci in the scanned images, the number of foci was normalized to a 20 mm^2^ area.

### Immunohistochemistry (IHC)

Hearts and livers were collected on day 21 from animals immunized with BCKD_k_ 111–130 and control groups (naive, CFA/PT, and RNase 43–56) and the tissues were examined for the presence of T cells, macrophages and granulocytes (neutrophils). To detect T cells, sections were stained with rabbit anti‐mouse CD3 (Abcam, Cambridge, MA); for macrophages, rabbit anti‐mouse CD11b (Abcam); for granulocytes, rat anti‐mouse Ly6G (Abcam) were used. Briefly, paraffin‐embedded heart sections were deparaffinized and rehydrated, and endogenous peroxidase activity was blocked with 3% hydrogen peroxide for 30 min. To retrieve antigens, sections were treated with 10 mM sodium citrate buffer (pH 6.0) in a water bath at 98°C for 15 min. After blocking for 30 min with 5% non‐fat dry milk, sections were incubated with primary antibodies at 4°C overnight. Sections were incubated with goat anti‐rabbit IgG or anti‐rat IgG, conjugated with HRP (Vector Laboratories, Burlingame, CA; and Abcam) as a secondary antibody, for 2 h at room temperature (RT) 2. After incubating with diaminobenzidine as a substrate, sections were fixed and counterstained with hematoxylin and examined as described above. For quantitative evaluation of CD3^+^, CD11b^+^, and Ly6G^+^ cells in the liver, random areas (5 to 13 mm^2^) from representative sections were blindly selected for each animal, and nuclear staining was confirmed using nuclear V9 software (Aperio Technologies, Vista, CA). Cells positive for each marker were then counted and normalized to a 1 mm^2^ area using Aperio ImageScope Analysis Software (Leica Biosystems, MN).

### Echocardiography and image analysis

Transthoracic echocardiography was performed in anesthetized (2% isoflurane, intranasal) mice on day 20 following immunization with BCKD_k_ 111–130. A research sonographer, blinded to the study groups, performed the measurements and data analysis. Closed‐chest imaging was performed in the short‐axis view at the mid‐LV level, verified by the presence of prominent papillary muscles, using a commercially available echocardiography system (Vivid 7, General Electric, Wauwatosa, WI) with an 11‐MHz M12‐L linear array transducer. Image depth was 1.5 cm, with acquisition of 293.6 frames/sec, second harmonic imaging and electrocardiographic gating. From the raw 2D image of the mid‐LV, anatomical M‐mode through the anteroseptal and inferolateral segments was used to measure the width of the intraventricular septum at diastole and the internal diameter of the LV at diastole and systole. End‐diastolic and end‐systolic volumes were calculated using the Tiechholz formula: LV Volume = [7/ (2.4 + LVID)] * LVID^3^. A cardiac cycle was defined from the peak of one R wave to the peak of the following R wave. Three consecutive heart beats were measured and the average was used for analysis.

### MHC class II (IA^k^)‐binding assay

To determine the affinities of peptides binding to IA^k^, soluble IA^k^ molecules expressed in the baculovirus/sf9 cells were used in the dissociation‐enhanced lanthanide fluoroimmunoassay (DELFIA) assay as we have described previously 22.

### MHC class II (IA^k^) dextramer staining

We created two dextramers (BCKD_k_ 111–130, specific; and RNase 43–56, control) to determine the antigen specificity of T cells sensitized with BCKD_k_ 111–130 and dextramer staining was performed as we have described previously 22. For staining T cells generated from livers, animals immunized with BCKD_k_ 111–130 twice as described above were euthanized on day 25 postimmunization, and livers collected after perfusions were homogenized and digested with type IV collagenase (400 U/ml; Worthington, Lakewood, NJ). MNCs were then harvested using 40%/75% percoll gradient centrifugation procedure 22. After stimulating with BCKD_k_ 111–130 for 2 days, cells were rested in IL‐2 medium and stained with dextramers on day 8 poststimulation.

### Cytokine bead array analysis

Groups of A/J mice were immunized with BCKD_k_ 111–130, and after 10 days animals were euthanized to prepare LNCs. Similarly LNCs were obtained on day 21 postimmunization from BCKD_k_ 71–90 and BCKD_k_ 141–160 immunized animals. Cells were stimulated with or without immunizing peptide or RNase 43–56 as control (50 µg/ml). Culture supernatants were collected on day 3 poststimulation and analyzed by cytokine bead array analysis as recommended by the manufacturer (BD Biosciences, Santiago, CA) 2, 35.

### Induction of EAM by adoptive transfer of antigen‐sensitized T cells

Groups of mice were immunized with BCKD_k_ 111–130 in CFA on days 0 and 7, and after 14 days, LNCs from these animals (2.5 × 10^6^ cells/ml) were stimulated with concanavalin‐A (2.5 µg/ml; Sigma–Aldrich, St. Louis, MO) for 2 days. Viable cells were administered (50–60 × 10^6^ cells/animal) into naive mice primed with LPS (25 µg/mouse; i.p. on day −4 and day 0) 2, 23, 24. Animals also received PT (100 ng/mouse) i.p. on day 0 and day 2 posttransfer. Mice that received saline or LPS/PT alone served as controls. At termination on day 14, tissues were collected for histology.

### Statistics

Student's *t*‐test was used to determine differences between groups for inflammatory foci, number of CD3^+^ and CD11b^+^ cells, T cell responses, echocardiography parameters, and cytokines. In determining T cell responses for some peptides with varied background levels, cpm values were scaled within the replicates and doses, using a constant multiplier determined by the average cpm values 2, 30. *p* ≤ 0.05 was considered significant.

## Conflict of Interest

The authors declare no financial or commercial conflicts of interest.

## Supporting information

Additional supporting information may be found in the online version of this article at the publisher's web‐site.

**Figure S1.** Evaluation of cardiac abnormalities in A/J mice immunized with BCKD_k_ 111‐130. Groups of mice were immunized with or without BCKD_k_ 111‐130; after 20 days, animals were euthanized, hearts were weighed, and the heart weight (wt) to body wt ratios were then determined. Mean ± SEM values representing heart and body wts and heart wt/body wt ratios for a group of mice are shown (*n *= 3/group). The p values were determined using Student's t‐test (*p < 0.05 and **p < 0.001)
**Figure S2**. BCKD_k_ peptides that induce T cell responses, but not myocarditis in immunized animals. Groups of A/J mice were immunized with the indicated peptides in CFA, and after three weeks, animals were euthanized to prepare LNCs from the draining lymph nodes. Cells were stimulated with the immunizing peptides or RNase 43‐56 (control) for two days, and after pulsing with tritiated‐thymidine for 16 h, proliferative responses were measured as cpm. Mean ± SEM values obtained from two individual experiments, each representing 2–3 mice, are shown. The p values were determined using Student's *t*‐test (*p < 0.05 and **p < 0.001 between indicated doses).
**Figure S3**. Proliferative responses of lymphocytes from naive animals to BCKD_k_ peptides. Lymphocytes containing a mixture of LNCs and splenocytes obtained from naive A/J mice were stimulated with the indicated peptides or RNase 43‐56 (control) for 2 days, and after pulsing with tritiated‐thymidine for 16 h, proliferative responses were measured as cpm. Mean ± SEM values obtained from five mice are shown.
**Figure S4.** Cytokine responses induced by BCKD_k_ 71‐90 and BCKD_k_ 141‐160. LNCs were prepared from animals that received CFA/peptide emulsions twice, and the cells were stimulated with or without immunizing peptides or RNase 43‐56 (control). Supernatants 2 collected on day 3 poststimulation were analyzed using cytokine‐capture beads and detection antibodies based on cytometric bead array analysis. Left panel A): BCKD_k_ 71‐90; right panel B): BCKD_k_ 141‐160. Mean ± SEM values obtained from three to four individual experiments are shown. The p values were determined using Student's *t*‐test (*p < 0.05, **p < 0.005 and ***p < 0.0005). ND; not detectable.
**Table S1**. List of overlapping peptides of BCKD_k_ used to determine their immunogenicity.
**Table S2**. T cell responses induced by peptides of BCKD_k_.
**Table S3**. Echocardiographic assessment of cardiac abnormalities in mice immunized with BCKD_k_ 111‐130.

## References

[1] Brown, C. A. , and J. B. O'Connell . 1995 Myocarditis and idiopathic dilated cardiomyopathy. Am. J. Med 99:309–314. 7653492 10.1016/S0002-9343(99)80164-8 PMC7119456

[2] Basavalingappa, R. H. , C. Massilamany , B. Krishnan , A. Gangaplara , G. Kang , V. K. Sharghi , Z. Han , S. Othman , Q. Li , J. J. Riethoven , et al. 2016 Identification of an Epitope from adenine nucleotide translocator 1 that induces inflammation in heart in A/J mice. Am. J. Pathol. 186:3160–3175. 27876151 10.1016/j.ajpath.2016.08.005 PMC5225289

[3] Ansari, A. A. , N. Neckelmann , F. Villinger , P. Leung , D. J. Danner , S. S. Brar , S. Zhao , M. B. Gravanis , A. Mayne , and M. E. Gershwin . 1994 Epitope mapping of the branched chain alpha‐ketoacid dehydrogenase dihydrolipoyl transacylase (BCKD‐E2) protein that reacts with sera from patients with idiopathic dilated cardiomyopathy. J. Immunol. 153:4754–4765. 7963542

[4] Caforio, A. L. , N. J. Mahon , and W. J. McKenna . 2001 Cardiac autoantibodies to myosin and other heart‐specific autoantigens in myocarditis and dilated cardiomyopathy. Autoimmunity 34:199–204. 11908778 10.3109/08916930109007385

[5] De Scheerder, I. K. , M. De Buyzere , J. Delanghe , A. Maas , D. L. Clement , and R. Wieme . 1991 Humoral immune response against contractile proteins (actin and myosin) during cardiovascular disease. Eur. Heart J. 12:88–94. 10.1093/eurheartj/12.suppl_d.88 1915462

[6] Gauntt, C. J. , H. M. Arizpe , A. L. Higdon , M. M. Rozek , R. Crawley , and M. W. Cunningham . 1991 Anti‐Coxsackievirus B3 neutralizing antibodies with pathological potential. Eur. Heart J. 12:124–129. 10.1093/eurheartj/12.suppl_d.124 1717270

[7] Kaya, Z. , S. Goser , S. J. Buss , F. Leuschner , R. Ottl , J. Li , M. Volkers , S. Zittrich , G. Pfitzer , N. R. Rose , et al. 2008 Identification of cardiac troponin I sequence motifs leading to heart failure by induction of myocardial inflammation and fibrosis. Circulation 118:2063–2072. 18955666 10.1161/CIRCULATIONAHA.108.788711 PMC2774217

[8] Chuang, D. T. 1989 Molecular studies of mammalian Branched‐Chain α‐Keto acid dehydrogenase complexes: domain structures, expression, and inborn errorsa. Ann. N. Y. Acad. Sci. 573:137–154. 2699394 10.1111/j.1749-6632.1989.tb14992.x

[9] Machius, M. , J. L. Chuang , R. M. Wynn , D. R. Tomchick , and D. T. Chuang . 2001 Structure of rat BCKD kinase: nucleotide‐induced domain communication in a mitochondrial protein kinase. Proc. Natl. Acad. Sci. U. S. A. 98:11218–11223. 11562470 10.1073/pnas.201220098 PMC58710

[10] Paxton, R. , and R. A. Harris . 1984 Regulation of branched‐chain alpha‐ketoacid dehydrogenase kinase. Arch. Biochem. Biophys. 231:48–57. 6721501 10.1016/0003-9861(84)90361-8

[11] Pettit, F. H. , S. J. Yeaman , and L. J. Reed . 1978 Purification and characterization of branched chain alpha‐keto acid dehydrogenase complex of bovine kidney. Proc. Natl. Acad. Sci. U. S. A. 75:4881–4885. 283398 10.1073/pnas.75.10.4881 PMC336225

[12] Ansari, A. , Y. Wang , D. Danner , M. Gravanis , A. Mayne , N. Neckelmann , K. Sell , and A. Herskowitz . 1991 Abnormal expression of histocompatibility and mitochondrial antigens by cardiac tissue from patients with myocarditis and dilated cardiomyopathy. Am. J. Pathol. 139:337. 1867322 PMC1886070

[13] Neumann, D. , N. Rose , A. Ansari , and A. Herskowitz . 1994 Induction of multiple heart autoantibodies in mice with coxsackievirus B3‐and cardiac myosin‐induced autoimmune myocarditis. J. Immunol. 152:343–350. 8254202

[14] Huang, Y. , M. Zhou , H. Sun , and Y. Wang . 2011 Branched‐chain amino acid metabolism in heart disease: an epiphenomenon or a real culprit? Cardiovasc. Res. 90:220–223. 21502372 10.1093/cvr/cvr070 PMC3078803

[15] Muller, E. A. , and D. J. Danner . 2004 Tissue‐specific translation of murine branched‐chain α‐ketoacid dehydrogenase kinase mRNA is dependent upon an upstream open reading frame in the 5′‐untranslated region. J. Biol. Chem. 279:44645–44655. 15302860 10.1074/jbc.M406550200

[16] Popov, K. M. , Y. Zhao , Y. Shimomura , J. Jaskiewicz , N. Y. Kedishvili , J. Irwin , G. W. Goodwin , and R. A. Harris . 1995 Dietary control and tissue specific expression of branched‐chain α‐ketoacid dehydrogenase kinase. Arch. Biochem. Biophys. 316:148–154. 7840610 10.1006/abbi.1995.1022

[17] Doering, C. B. , C. Coursey , W. Spangler , and D. J. Danner . 1998 Murine branched chain α‐ketoacid dehydrogenase kinase; cDNA cloning, tissue distribution, and temporal expression during embryonic development. Gene 212:213–219. 9611264 10.1016/s0378-1119(98)00182-6

[18] Ævarsson, A. , J. L. Chuang , R. M. Wynn , S. Turley , D. T. Chuang , and W. G. Hol . 2000 Crystal structure of human branched‐chain α‐ketoacid dehydrogenase and the molecular basis of multienzyme complex deficiency in maple syrup urine disease. Structure. 8:277–291. 10745006 10.1016/s0969-2126(00)00105-2

[19] Pummerer, C. L. , K. Luze , G. Grässl , K. Bachmaier , F. Offner , S. K. Burrell , D. M. Lenz , T. J. Zamborelli , J. M. Penninger , and N. Neu . 1996 Identification of cardiac myosin peptides capable of inducing autoimmune myocarditis in BALB/c mice. J. Clin. Invest. 97:2057. 8621795 10.1172/JCI118642 PMC507280

[20] Howell, C. D. , and T. D. Yoder . 1994 Murine experimental autoimmune hepatitis: nonspecific inflammation due to adjuvant oil. Clin. Immunol. Immunopathol. 72:76–82. 8020196 10.1006/clin.1994.1109

[21] Massilamany, C. , A. Gangaplara , D. Steffen , and J. Reddy . 2011 Identification of novel mimicry epitopes for cardiac myosin heavy chain‐α that induce autoimmune myocarditis in A/J mice. Cell. Immunol. 271:438–449. 21939961 10.1016/j.cellimm.2011.08.013

[22] Massilamany, C. , B. Upadhyaya , A. Gangaplara , C. Kuszynski , and J. Reddy . 2011 Detection of autoreactive CD4 T cells using major histocompatibility complex class II dextramers. BMC. Immunol. 12:1. 21767394 10.1186/1471-2172-12-40 PMC3151213

[23] Hamada, Y. , M. Takata , H. Kiyoku , H. Enzan , Y. Doi , and S. Fujimoto . 2004 Monomethoxypolyethylene glycol‐modified cardiac myosin treatment blocks the active and passive induction of experimental autoimmune myocarditis. Circ. J. 68:149–155. 14745151 10.1253/circj.68.149

[24] Massilamany, C. , R. H. Basavalingappa , R. A. Rajasekaran , V. Khalilzad‐Sharghi , Z. Han , S. Othman , D. Steffen , and J. Reddy . 2016 Localization of CD8 T cell epitope within cardiac myosin heavy chain‐α 334–352 that induces autoimmune myocarditis in A/J mice. Int. J. Cardiol. 202:311–321. 26422020 10.1016/j.ijcard.2015.09.016 PMC4656055

[25] Miller, S. D. , C. L. Vanderlugt , W. S. Begolka , W. Pao , R. L. Yauch , K. L. Neville , Y. Katz‐Levy , A. Carrizosa , and B. S. Kim . 1997 Persistent infection with Theiler's virus leads to CNS autoimmunity via epitope spreading. Nat. Med. 3:1133–1136. 9334726 10.1038/nm1097-1133

[26] Göser, S. , M. Andrassy , S. J. Buss , F. Leuschner , C. H. Volz , R. Öttl , S. Zittrich , N. Blaudeck , S. E. Hardt , and G. Pfitzer . 2006 Cardiac troponin I but not cardiac troponin T induces severe autoimmune inflammation in the myocardium. Circulation 114:1693–1702. 17015788 10.1161/CIRCULATIONAHA.106.635664

[27] Kaya, Z. , H. A. Katus , and N. R. Rose . 2010 Cardiac troponins and autoimmunity: their role in the pathogenesis of myocarditis and of heart failure. Clin. Immunol. 134:80–88. 19446498 10.1016/j.clim.2009.04.008 PMC2819185

[28] Yüksel, M. , D. Laukens , F. Heindryckx , H. V. Vlierberghe , A. Geerts , F. Wong , L. Wen and I. Colle . 2014 Hepatitis mouse models: from acute‐to‐chronic autoimmune hepatitis. Int. J. Exp. Pathol. 95:309–320. 25112417 10.1111/iep.12090 PMC4209923

[29] Bettelli, E. , M. Oukka , and V. K. Kuchroo . 2007 TH‐17 cells in the circle of immunity and autoimmunity. Nat. Immunol. 8:345–350. 17375096 10.1038/ni0407-345

[30] Massilamany, C. , O. A. Asojo , A. Gangaplara , D. Steffen , and J. Reddy . 2011 Identification of a second mimicry epitope from Acanthamoeba castellanii that induces CNS autoimmunity by generating cross‐reactive T cells for MBP 89–101 in SJL mice. Int. Immunol. 23:729–739. 22058327 10.1093/intimm/dxr084

[31] Beland, K. , P. Lapierre , I. Djilali‐Saiah , and F. Alvarez . 2012 Liver restores immune homeostasis after local inflammation despite the presence of autoreactive T cells. PLoS ONE 7:e48192. 23110209 10.1371/journal.pone.0048192 PMC3480501

[32] Leung, P. S. , O. Park , K. Tsuneyama , M. J. Kurth , K. S. Lam , A. A. Ansari , R. L. Coppel , and M. E. Gershwin . 2007 Induction of primary biliary cirrhosis in guinea pigs following chemical xenobiotic immunization. J. Immunol. 179:2651–2657. 17675529 10.4049/jimmunol.179.4.2651

[33] Wakabayashi, K. , Z. X. Lian , P. S. Leung , Y. Moritoki , K. Tsuneyama , M. J. Kurth , K. S. Lam , K. Yoshida , G. X. Yang , T. Hibi , et al. 2008 Loss of tolerance in C57BL/6 mice to the autoantigen E2 subunit of pyruvate dehydrogenase by a xenobiotic with ensuing biliary ductular disease. Hepatology 48:531–540. 18563844 10.1002/hep.22390 PMC3753011

[34] Baker, M. J. , C. S. Palmer , and D. Stojanovski . 2014 Mitochondrial protein quality control in health and disease. Br. J. Pharmacol. 171:1870–1889. 24117041 10.1111/bph.12430 PMC3976610

[35] Jia, T. , A. Anandhan , C. Massilamany , R. A. Rajasekaran , R. Franco , and J. Reddy . 2015 Association of autophagy in the cell death mediated by dihydrotestosterone in autoreactive t cells independent of antigenic stimulation. J. Neuroimmune Pharmacol. 10:620–634. 26416183 10.1007/s11481-015-9633-x PMC4662616

