# Supplementary material for: Branched chain α‐ketoacid dehydrogenase kinase 111–130, a T cell epitope that induces both autoimmune myocarditis and hepatitis in A/J mice
Source: Immun Inflamm Dis. 2017 Jun 9;5(4):421–34. doi: 10.1002/iid3.177 (PMC5691315; doi:10.1002/iid3.177)
Supplement: Supplementary file 1 — Figure S1. Evaluation of cardiac abnormalities in A/J mice immunized with BCKDk 111‐130. Groups of mice were immunized with or without BCKDk 111‐130; after 20 days, animals were euthanized, hearts were weighed, and the heart weight (wt) to body wt ratios were then determined. Mean ± SEM values representing heart and body wts and heart wt/body wt ratios for a group of mice are shown (n = 3/group). The p values were determined using Student's t‐test (*p < 0.05 and **p < 0.001) Figure S2. BCKDk peptides that induce T cell responses, but not myocarditis in immunized animals. Groups of A/J mice were immunized with the indicated peptides in CFA, and after three weeks, animals were euthanized to prepare LNCs from the draining lymph nodes. Cells were stimulated with the immunizing peptides or RNase 43‐56 (control) for two days, and after pulsing with tritiated‐thymidine for 16 h, proliferative responses were measured as cpm. Mean ± SEM values obtained from two individual experiments, each representing 2–3 mice, are shown. The p values were determined using Student's t‐test (*p < 0.05 and **p < 0.001 between indicated doses). Figure S3. Proliferative responses of lymphocytes from naive animals to BCKDk peptides. Lymphocytes containing a mixture of LNCs and splenocytes obtained from naive A/J mice were stimulated with the indicated peptides or RNase 43‐56 (control) for 2 days, and after pulsing with tritiated‐thymidine for 16 h, proliferative responses were measured as cpm. Mean ± SEM values obtained from five mice are shown. Figure S4. Cytokine responses induced by BCKDk 71‐90 and BCKDk 141‐160. LNCs were prepared from animals that received CFA/peptide emulsions twice, and the cells were stimulated with or without immunizing peptides or RNase 43‐56 (control). Supernatants 2 collected on day 3 poststimulation were analyzed using cytokine‐capture beads and detection antibodies based on cytometric bead array analysis. Left panel A): BCKDk 71‐90; right panel B): BCKDk 141‐160. M [file IID3-5-421-s001.pdf]

10. Supporting Information

10.1. Figure S1: Evaluation of cardiac abnormalities in A/J mice immunized with $BCKD_k$ 111-130. Groups of mice were immunized with or without $BCKD_k$ 111-130; after 20 days, animals were euthanized, hearts were weighed, and the heart weight (wt) to body wt ratios were then determined. Mean ± SEM values representing heart and body wts and heart wt/body wt ratios for a group of mice are shown (n=3/group). The *p* values were determined using Student's *t*-test ($*P < 0.05$ and $**P < 0.001$).

10.2. Figure S2: $BCKD_k$ peptides that induce T cell responses, but not myocarditis in immunized animals. Groups of A/J mice were immunized with the indicated peptides in CFA, and after three weeks, animals were euthanized to prepare LNCs from the draining lymph nodes. Cells were stimulated with the immunizing peptides or RNase 43-56 (control) for two days, and after pulsing with tritiated-thymidine for 16 hours, proliferative responses were measured as cpm. Mean ± SEM values obtained from two individual experiments, each representing 2 to 3 mice, are shown. The *p* values were determined using Student's *t*-test ($*P < 0.05$ and $**P < 0.001$ between indicated doses).

10.3. Figure S3: Proliferative responses of lymphocytes from naive animals to $BCKD_k$ peptides. Lymphocytes containing a mixture of LNCs and splenocytes obtained from naive A/J mice were stimulated with the indicated peptides or RNase 43-56 (control) for two days, and after pulsing with tritiated-thymidine for 16 hours, proliferative responses were measured as cpm. Mean ± SEM values obtained from five mice are shown.

10.4. Figure S4: Cytokine responses induced by $BCKD_k$ 71-90 and $BCKD_k$ 141-160. LNCs were prepared from animals that received CFA/peptide emulsions twice, and the cells were stimulated with or without immunizing peptides or RNase 43-56 (control). Supernatants

collected on day 3 poststimulation were analyzed using cytokine-capture beads and detection antibodies based on cytometric bead array analysis. Left panel **A)**: $BCKD_k$ 71-90; right panel **B)**: $BCKD_k$ 141-160. Mean ± SEM values obtained from three to four individual experiments are shown. The *p* values were determined using Student's *t*-test (*$P < 0.05$, **$P < 0.005$ and ***$P < 0.0005$). ND; not detectable.

Table S1: List of overlapping peptides of $BCKD_k$ used to determine their immunogenicity

Peptide	Sequence
$BCKD_K$ 1-20	STSATDTHHVELARERSKTV
$BCKD_K$ 11-30	ELARERSKTVTSFYNQSAID
$BCKD_K$ 21-40	TSFYNQSAIDVAAEKPSVRL
$BCKD_K$ 31-50	VAAEKPSVRLTPTMMLYSGR
$BCKD_K$ 41-60	TPTMMLYSGRSQDGSHLLKS
$BCKD_K$ 51-70	SQDGSHLLKSGRYLQQELPV
$BCKD_K$ 61-80	GRYLQQELPVRIAHRIKGFR
$BCKD_K$ 71-90	RIAHRIKGFRSLPFIIGCNP
$BCKD_K$ 81-100	SLPFIIGCNPTILHVHELYI
$BCKD_K$ 91-110	TILHVHELYIRAFQKLTDFP
$BCKD_K$ 101-120	RAFQKLTDFPPIKDQADEAQ
$BCKD_K$ 111-130	PIKDQADEAQYCQLVRQLLD
$BCKD_K$ 121-140	YCQLVRQLLDDHKDVVTLLA
$BCKD_K$ 131-150	DHKDVVTLLAEGLRESRKHI
$BCKD_K$ 141-160	EGLRESRKHIQDEKLVRYFL
$BCKD_K$ 151-170	QDEKLVRYFLDKTLTSRLGI
$BCKD_K$ 161-180	DKTLTSRLGIRMLATHHLAL
$BCKD_K$ 171-190	RMLATHHLALHEDKPDFVGI
$BCKD_K$ 181-200	HEDKPDFVGIICTRLSPKKI
$BCKD_K$ 191-210	ICTRLSPKKIIEKWVDFARR
$BCKD_K$ 201-220	IEKWVDFARRLCEHKYGNAP
$BCKD_K$ 211-230	LCEHKYGNAPRVRINGHVAA
$BCKD_K$ 221-240	RVRINGHVAARFPFIPMPLD
$BCKD_K$ 231-250	RFPFIPMPLDYILPELLKNA
$BCKD_K$ 241-260	YILPELLKNAMRATMESHLD
$BCKD_K$ 251-270	MRATMESHLDTPYNVPDVVI
$BCKD_K$ 261-280	TPYNVPDVVITIANNDIDLI
$BCKD_K$ 271-290	TIANNDIDLIIRISDRGGGI
$BCKD_K$ 281-300	IRISDRGGGIAHKDLDRVMD
$BCKD_K$ 291-310	AHKDLDRVMDYHFTTAEAST
$BCKD_K$ 301-320	YHFTTAEASTQDPRINPLFG
$BCKD_K$ 311-330	QDPRINPLFGHLDMHSGGQS
$BCKD_K$ 321-340	HLDMHSGGQSGPMHGFGFGL
$BCKD_K$ 331-350	GPMHGFGFGLPTSRAYAEYL
$BCKD_K$ 341-360	PTSRAYAEYLGGSLQLQSLQ
$BCKD_K$ 351-370	GGSLQLQSLQGIGTDVYLRL
$BCKD_K$ 361-380	GIGTDVYLRLRHIDGREESF

Table S2: T cell responses induced by peptides of $BCKD_k$

Pools	Peptides	Fold difference†	
		10 (μg/μl)	100 (μg/μl)
I	RNase 43-56 (control)	0.93	0.99
	$BCKD_k$ 1-20	0.97	1.18
	$BCKD_k$ 11-30	0.86	1.16
	$BCKD_k$ 21-40	0.82	1.35
	**$BCKD_k$ 31-50**	**1.16**	**1.34**
	$BCKD_k$ 41-60	1.06	1.21
II	RNase 43-56 (control)	1.04	1.08
	**$BCKD_k$ 51-70**	**1.28**	**1.47**
	**$BCKD_k$ 61-80**	**1.46**	**1.80**
	**$BCKD_k$ 71-90**	**1.49**	**2.55**
	$BCKD_k$ 81-100	1.08	1.81
	**$BCKD_k$ 91-110**	**1.86**	**2.05**
III	RNase 43-56 (control)	1.04	1.06
	**$BCKD_k$ 101-120**	**1.21**	**1.46**
	**$BCKD_k$ 111-130**	**1.21**	**1.75**
	**$BCKD_k$ 121-140**	**1.41**	**1.84**
	$BCKD_k$ 131-150	1.08	1.53
	**$BCKD_k$ 141-160**	**1.58**	**1.66**
IV	RNase 43-56 (control)	1.05	1.12
	$BCKD_k$ 151-170	1.08	1.46
	**$BCKD_k$ 161-180**	**1.24**	**1.88**
	$BCKD_k$ 171-190	0.98	1.12
	$BCKD_k$ 181-200	1.03	1.22
	$BCKD_k$ 191-210	1.14	1.40
V	RNase 43-56 (control)	0.92	0.97
	**$BCKD_k$ 201-220**	**1.18**	**1.17**
	**$BCKD_k$ 211-230**	**1.17**	**1.22**
	**$BCKD_k$ 221-240**	**1.13**	**1.26**
	**$BCKD_k$ 231-250**	**1.37**	**1.64**
	**$BCKD_k$ 241-260**	**1.11**	**1.28**
VI	RNase 43-56 (control)	0.88	1.10
	$BCKD_k$ 251-270	1.12	1.23
	**$BCKD_k$ 261-280**	**1.51**	**1.41**
	**$BCKD_k$ 271-290**	**1.36**	**1.74**
	$BCKD_k$ 281-300	1.16	1.18
VII	RNase 43-56 (control)	1.16	1.24
	**$BCKD_k$ 291-310**	**2.04**	**3.02**
	**$BCKD_k$ 301-320**	**1.89**	**2.21**
	$BCKD_k$ 311-330	1.05	1.46
	**$BCKD_k$ 321-340**	**1.49**	**2.15**
VIII	RNase 43-56 (control)	1.00	1.25
	**$BCKD_k$ 331-350**	**1.63**	**1.65**
	**$BCKD_k$ 341-360**	**1.51**	**1.57**
	**$BCKD_k$ 351-370**	**1.39**	**1.59**
	$BCKD_k$ 361-380	1.21	1.25

†Represents fold difference in T cell proliferative responses induced with $BCKD_k$ peptides in relation to medium controls. Significant differences for $BCKD_k$ peptides vs. RNase 43-56 are bolded ($P \leq 0.005$).

Table S3: Echocardiographic assessment of cardiac abnormalities in mice immunized with $BCKD_k$ 111-130

Parameters	Naïve	$BCKD_k$ 111-130
Interventricular septal thickness at diastole (mm)	0.70 ± 0.06	0.93 ± 0.03*
LV internal diameter at diastole (mm)	3.50 ± 0.03	3.20 ± 0.06
End diastolic volume (μl)	110.00 ± 3.33	86.60 ± 3.33
End systolic volume (μl)	30.00 ± 10.00	20.00 ± 0.00

Data represent mean ± SEM with *$P < 0.05$ vs. naïve group.

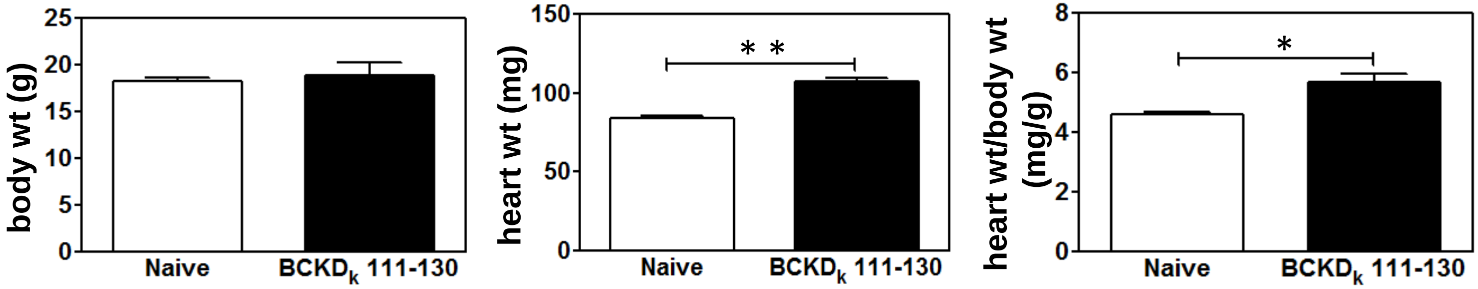

Figure S1: Evaluation of cardiac abnormalities in A/J mice immunized with $BCKD_k$ 111-130

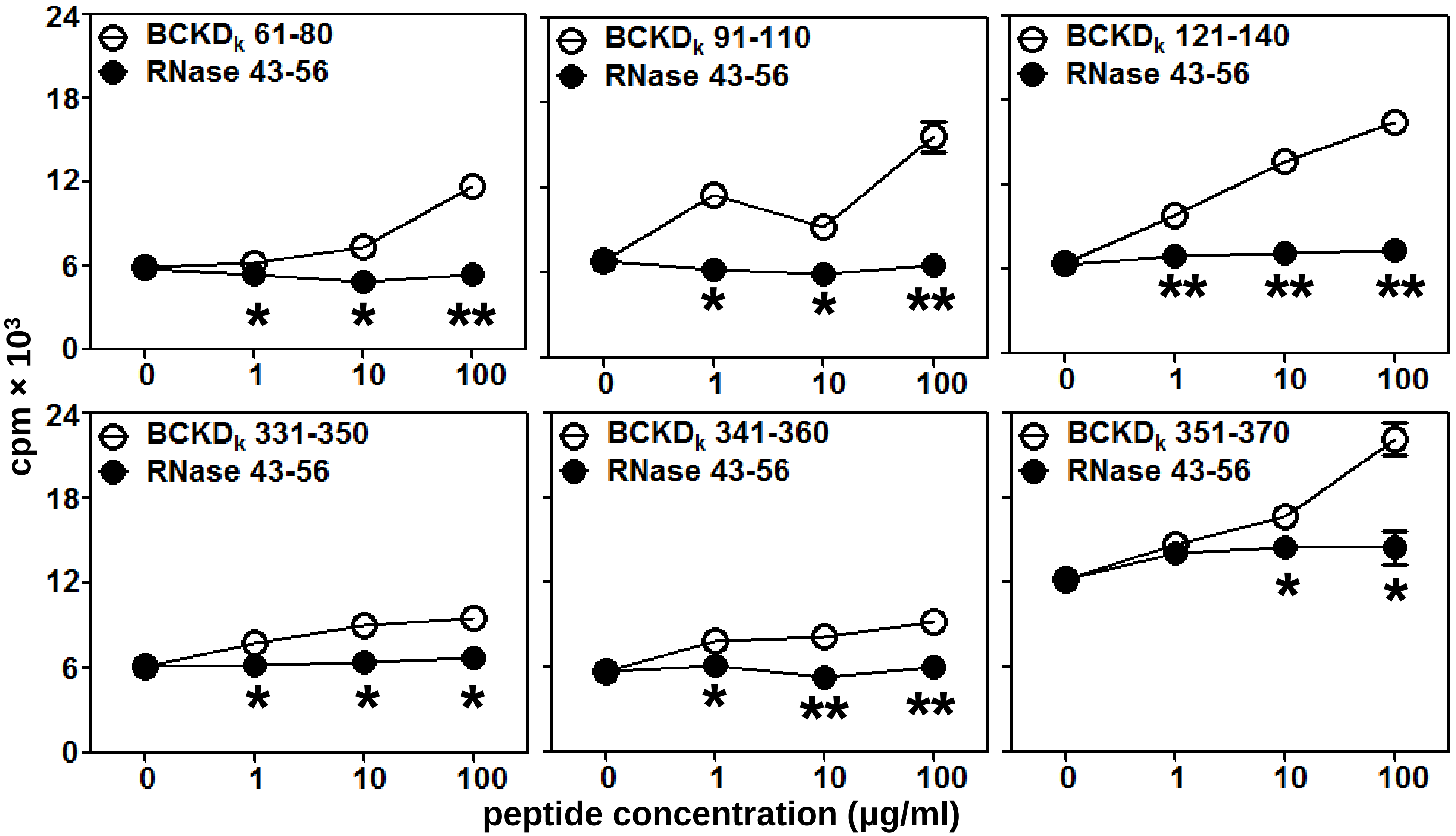

Figure S2: BCKD$_k$ peptides that induce T cell responses, but not myocarditis in immunized animals

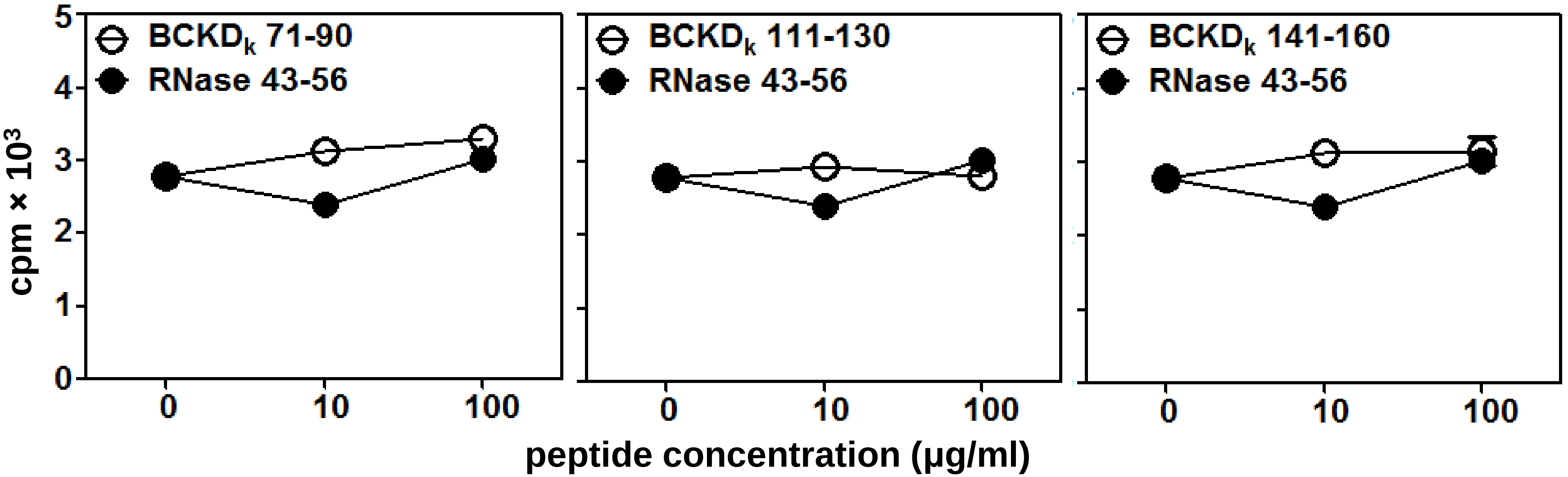

Figure S3: Proliferative responses of lymphocytes from naïve animals to BCKD$_k$ peptides

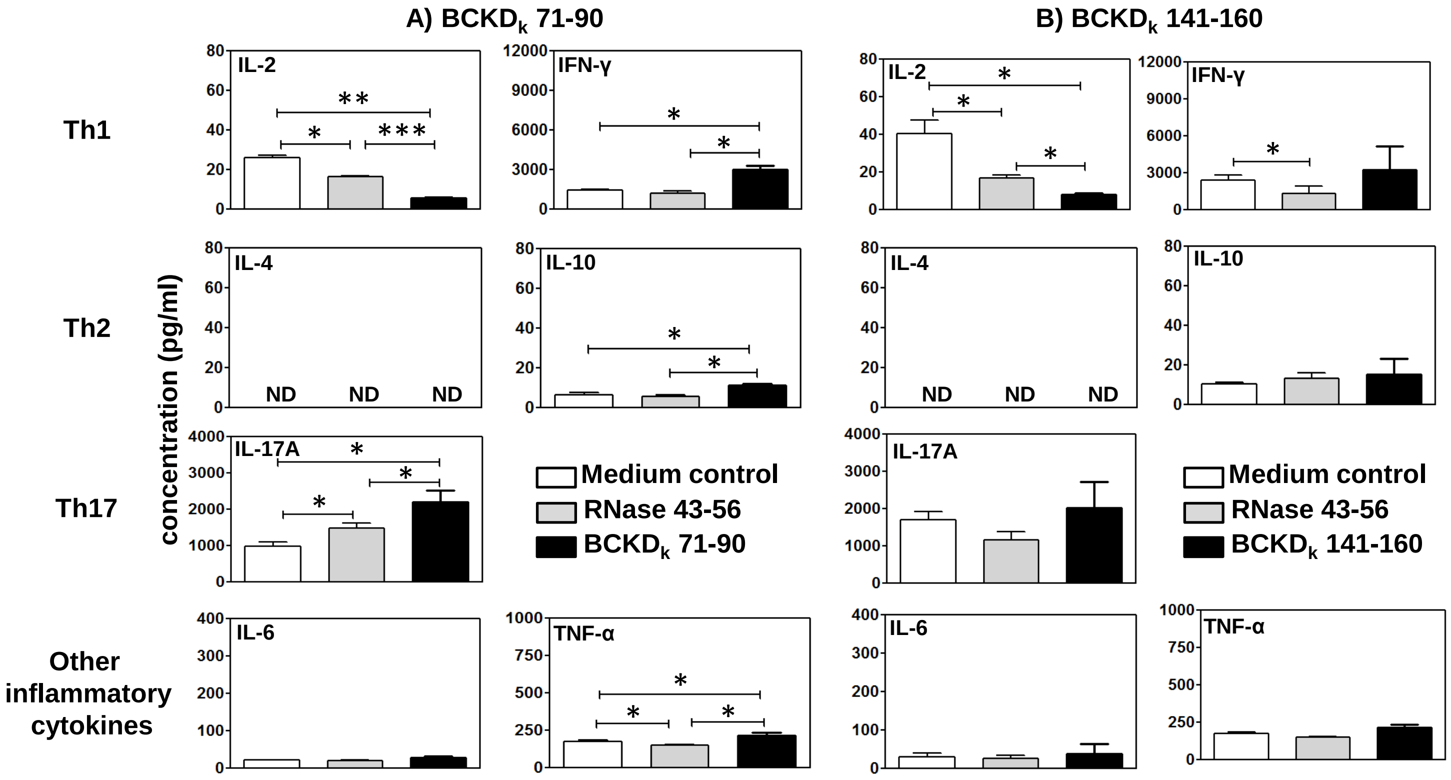

Figure S4: Cytokine responses induced by BCKD$_k$ 71-90 and BCKD$_k$ 141-160
